# Death of Pereira

**Published:** 1853-08

**Authors:** 


*Death of Dr. Pereira.*

We are permitted to extract the following reference to the death of this distinguished physician from a private letter to Dr. Duck of this city. The writer says: "Poor Pereria, his loss is felt by all; he was buried at the Kensalfreen cemetry, and was followed by the whole school of the London Hospital and also by between thirty and forty practitioners, many of them, including myself, coming a distance of between six and seven miles. Dr. Billing kindly took me in his carriage coming home, when I had the advantage of hearing his opinion as to the cause of death. Dr. B. thinks that when Dr. Pereria slipped on the back stair case of the College of Surgeons, in addition to the rupture of the tendon of the left quadriceps extensor femoris, he also ruptured the two inner coats of one of the sinuses, at the origin of the aorta, and that on exerting himself to relieve his attendants while lifting him into bed, a fortnight afterwards, he ruptured the remaining coat and died from internal hemorrhage. His friends objected to a post-mortem examination, and it was not urged. A committee of four or five physicians are completing the second part of the 2d volume of the learned Doctor's Materia Medica, which Dr. Billing says is a perfect encyclopedia. The greater part was already finished and in the press." J.

